# Photocatalytic degradation of microcystin-LR with a nanostructured photocatalyst based on upconversion nanoparticles@TiO_2_ composite under simulated solar lights

**DOI:** 10.1038/s41598-017-14746-6

**Published:** 2017-10-31

**Authors:** Shijia Wu, Jiajia Lv, Fang Wang, Nuo Duan, Qian Li, Zhouping Wang

**Affiliations:** 1 State Key Laboratory of Food Science and Technology, Jiangnan University, Wuxi, 214122 China; 2 School of Food Science and Technology, Jiangnan University, Wuxi, 214122 China

## Abstract

In this work, we prepared advanced upconversion nanoparticle coated with TiO_2_ photocatalysts (NaYF_4_:Yb,Tm@TiO_2_) to utilize not only UV energy but also the large portion of NIR energy in order to improve the utilization efficiency of solar lights. The MC-LR (10 μg/mL) degradation rate can be approached 100% within 30 min at the concentration of NaYF_4_:Yb,Tm@TiO_2_ 0.4 mg/mL and initial pH value 4, while 61%, using pure TiO_2_ (P25) under simulated solar lights. The reaction processes were studied and fitted with the pseudo-first-order kinetic model. Highly reactive hydroxyl radicals (•OH) were found to be the major reactive species. Meanwhile, seven degradation intermediates of MC-LR were examined by liquid chromatography/mass spectrometry and the degradation mechanism was analyzed. The main degradation pathways were proposed based on the molecular weight of the intermediates and the reaction mechanism are hydroxylation on the diene bonds and the aromatic ring of Adda. The products were evaluated to be nontoxic based on the construction of the intermediates. This study demonstrated that the NIR energy can be used as the driving source for photocatalysis besides the UV and the NIR-responsed photocatalysis had a high-efficiency and potential for MC-LR degradation.

## Introduction

Cyanobacterial blooms pose a serious threat to aquatic ecosystems due to the liberation of cyanotoxins into water sources. Microcystins (MCs) are cyclic polypeptides represent one of the most common cyanotoxins released by cyanobacterial in the water of eutrophication^1^. Microcystins are well known highly acute neurotoxins, hepatotoxins and cytotoxins, and the toxicity of MCs is assumed to the inhibition of the activities of protein phosphatases 1 and 2 A (PP1 and PP2A), two key enzymes in cellular processes^2,3^. Microcystins tend to persist in the aquatic environment for a long period of time, and not only can be accumulated into aquatic organisms, but also transferred to higher trophic levels through the biological chain^4^. The contamination of aquatic products (such as mussel and fish) with MCs has harmful impact on both ecosystems functioning and human health. Medical studies have shown that MCs would cause human liver organ lesions, tumors, and even cancer. Due to their toxicity, degradation and detoxification pathways of MCs have aroused growing concern.

In virtue of the cyclic structure and double bonds in the molecule, MCs are physic-chemically stable and recalcitrant to hydrolysis or disintegrate naturally even at high temperature or low pH^5^. Several removal techniques have been adopted for microcystins clarification, such as activated carbon adsorption^6,7^, membrane filtration^8,9^, ultrasonic method^10^, oxidative degradation^11–15^ and biodegradation^16,17^. It is worth noting that though adsorption and filtration could isolate MCs, the toxicity still retain due to intactness of the structure. In addition, chemistry degradation is mainly dependent on reagents increasingly. Meanwhile, the reagents will form contaminant to pollute the environment again. Biodegradation is a potential approach to solve toxin, and even is green pollution-free. The weakness is that it is usually to spend a long time, improving the efficiency is great to be concerned. Among of above methods, the photocatalytic destruction of cyanotoxins, particularly microcystin-LR, has been studied in detail and reported to be a very potential process^18–20^.

Photocatalytic technology has been constantly recognized as a promising green route (advanced oxidation processes, AOPs) for application in controlling pollutant and producing energy, such as environmental remediation and hydrogen production^21,22^. The free radicals formed subsequently have strong oxidizing ability and can mineralize organic pollutants to CO_2_ and H_2_O. Among various photocatalysts reported so far, titanium dioxide (TiO_2_) is undoubtedly the most widely used in the degradation of inorganic or organic pollutants due to its strong oxidizing power, extraordinary chemical stability, low cost and non-toxicity toward both human and environment^23–25^. However, this photocatalyst requires ultraviolet (UV) light to be activated because of its large bandgap of ∼3.2 eV. It is well known that the solar spectra compose of UV (290–400 nm), visible (400–760 nm) and infrared (IR) radiation (760–3000 nm), in which the corresponding solar energy is ~5% in the UV range, ~49% in the visible range and about 46% in the IR spectral range^26,27^. Solar energy has been widely regarded as one of the most promising renewable energy sources in the world since it is a free, non-polluting, inexhaustible resource. Regrettably, more than 90% of overall solar spectrum cannot be utilized to activate TiO_2_ for photocatalysis.

To resolve this problem, it is still a challenge to develop alternative TiO_2_ to extend the light absorption region to the visible and even NIR range and improve the catalytic efficiencies. Lanthanide-doped materials have become known as efficient luminescent materials due to their narrow emission bands, low toxicity, as well as their physical and chemical stability. Moreover, when doped with appropriate ions, they can convert NIR excitation light into visible or UV emission light^28,29^. Recently, there have been reports of the combination of upconversion luminescence (UC) agents with the semiconductors TiO_2_, which allowed the use of a visible or NIR light source for catalysis. For example, Yb^3+^/Tm^3+^ co-doped YF_3_ or NaYF_4_ UCNPs acts as a medium for converting NIR to UV and visible light via multiphoton upconversion processes under 980 nm excitation^30–32^. After absorbing NIR light, transfers energy to TiO_2_ to generate strongly oxidative holes (h^+^) and reductive electrons (e^-^) which are powerful oxidizing agents for environmental pollutants.

In the past decade, various photocatalysts, especially, TiO_2_-based materials were used to microcystins degradation. Most of the methods were also restricted in ultraviolet radiation. There are few reports about microcystins degradation utilizing visible or NIR light. In our work, the synthetized nanomicrospheres NaYF_4_:Yb^3+^,Tm^3+^@TiO_2_ core-shell structure is characterized by a series of characterization devices and then used to degrade microcystins under simulated sunlight because it is beneficial for TiO_2_ shells to absorb the UV light from the upconversion NCs. This research might provide clues to develop a highly efficient, convenient, safe, and cost-effective photocatalytic system applied in microcystins decontamination. Moreover, it is crucial to understand the potential mechanism for further improving efficiency of photocatalysis under the solar energy irradiation.

## Results

### Characterization of UCNP@TiO_2_ photocatalyst

**Figure 1:**
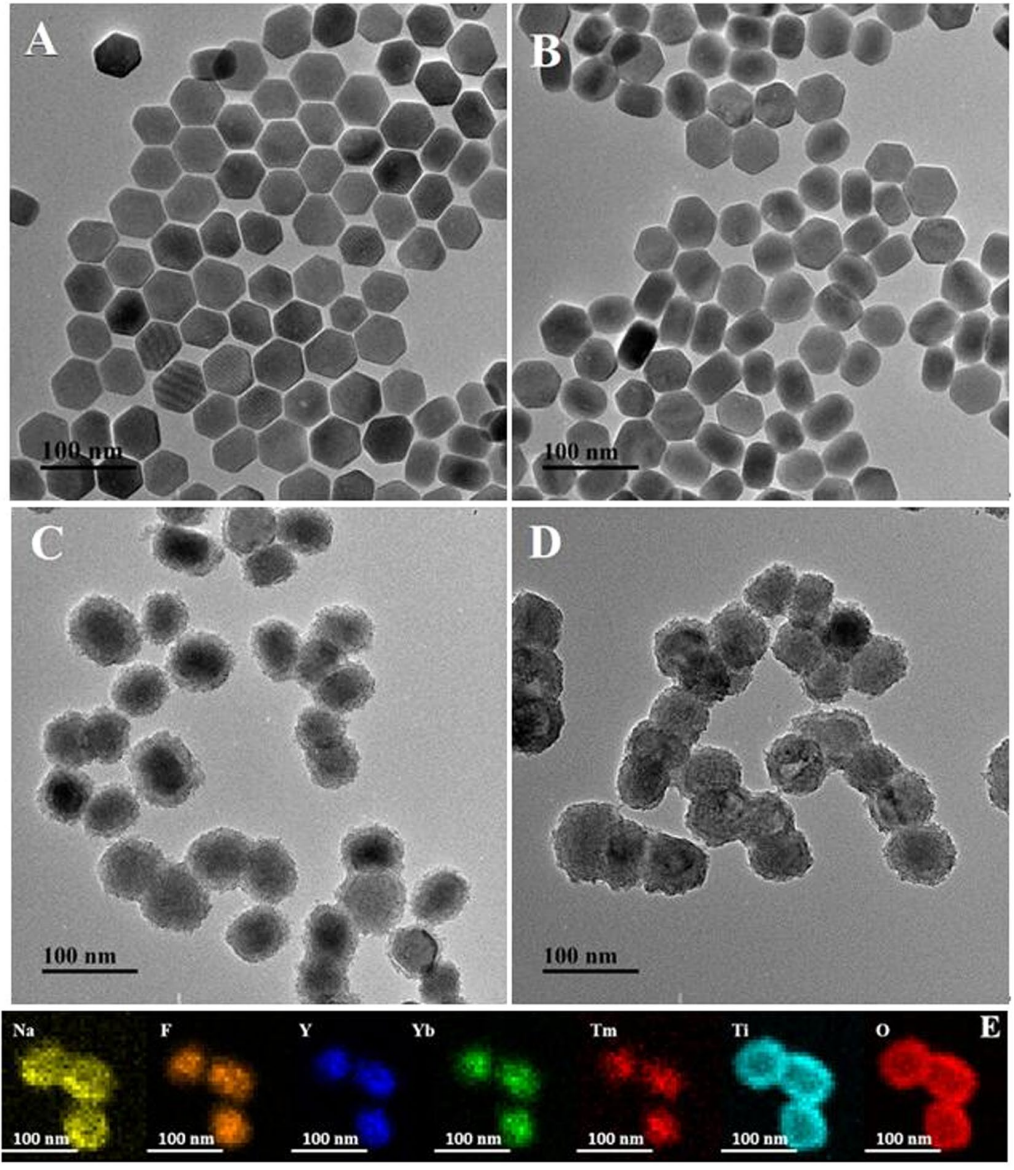
TEM images of NaYF_4_:Yb, Tm@TiO_2_ nanoparticles at different synthetic stages: NaYF_4_:Yb, Tm (**A**), NaYF_4_:Yb, Tm/CTAB (**B**), NaYF_4_:Yb, Tm@A-TiO_2_ (**C**)_,_ NaYF_4_:Yb, Tm@TiO_2_ after annealing (**D**). The element mapping of NaYF_4_:Yb,Tm@TiO_2_ nanoparticles.

Typical, TEM images of the samples from the bare UCNP to final UCNP@TiO_2_ nanocomposites were illustrated in Fig. 1. Uniform size of UCNP was obtained with a mean diameter of about 50 nm (Fig. 1A). After surface modification with a CTAB layer, size and morphology of the as-prepared samples remain unchanged by the aid of a thin CTAB molecules layer (Fig. 1B). In Fig. 1C, 15 nm uniform TiO_2_ shell deposited on the UCNP was subsequently examined exactly. Additionally, nanoporous are obtained in the shell after annealing the sample (Fig. 1D). Elemental mapping was employed to further examine the composition of the NaYF_4_:Yb,Tm@TiO_2_. The element mapping in Fig. 1E demonstrated that the samples consist of Ti, O, Na, Y, Yb, Tm and F, indicating that the obtained nanospheres were composed of the target materials. The result was consisted with the EDX spectrum of NaYF_4_:Yb,Tm@TiO_2_ nanospheres (Fig. S1). The X-ray photoelectron spectra (XPS) analysis was studied to understand surface composition and elemental chemical status of the UCNP@TiO_2_. The peaks of the binding energies at 283.5 eV, 157.1 eV, 682.2 eV and 1071.6 eV were attributed to C_1s_, Y_3d5/2_, F_1s_, and Na_1s_, respectively. Fig. S2 showed the characteristic peak of element O at 530 eV. Two XPS peaks at 457 eV and 464 eV are attribute to Ti^4+^ ions, corresponding to Ti^4+^_2p 3/2_, Ti^4+^_2p 1/2_, respectively. To investigate the crystal structure of the as-synthesized product, the X-ray diffraction (XRD) patterns of the as-formed NaYF_4_:Yb,Tm and UCNP@TiO_2_ nanoparticles were measured and the results were presented in Fig. S3. As shown in Fig. S3a, the position and relative intensity of all diffraction peaks can be readily indexed to the pure hexagonal-phase NaYF_4_ according to the JCPDS files NO.16-0334. Similarly, the characteristic diffraction peaks of TiO_2_ can be observed in Fig. S3b, corresponding to anatase titania according to the JCPDS files NO.21-1272.

**Figure 2:**
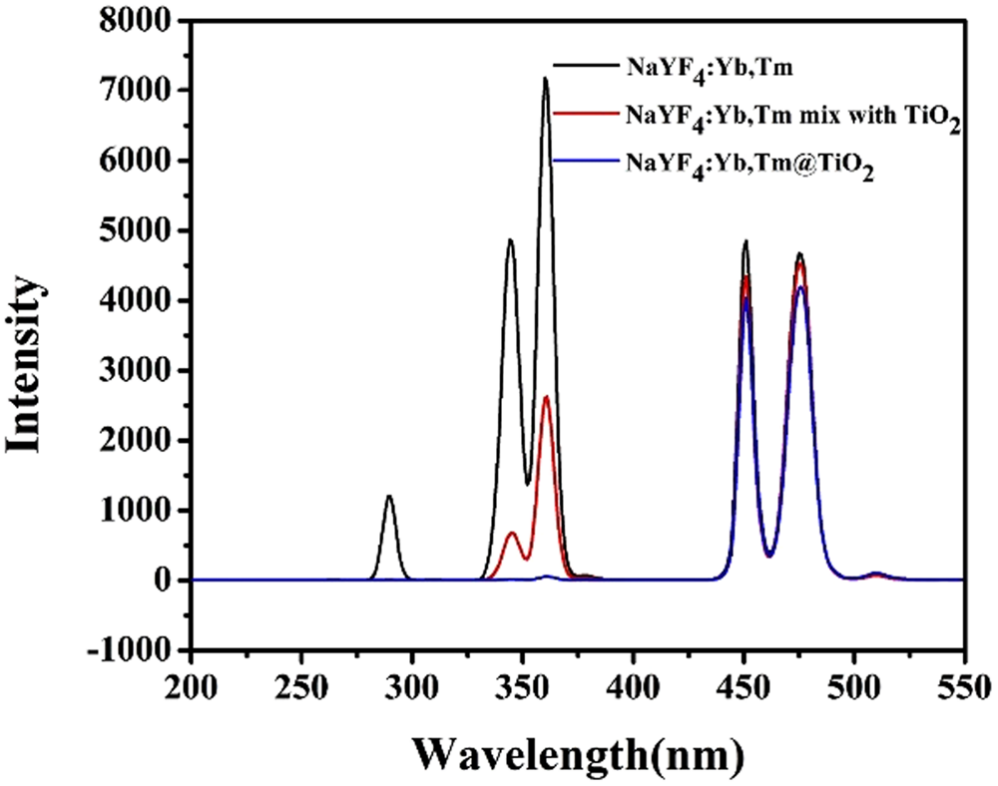
The fluorescence spectra of NaYF_4_:Yb, Tm, NaYF_4_:Yb, Tm-TiO_2_ mixture and NaYF_4_:Yb, Tm@TiO_2_ nanoparticles.

As above mentioned, it was confirmed that the core-shell UCNP@TiO_2_ composites were synthesized successfully relied on morphology and crystal structure. To illuminate the upconversion energy transformation, the emission spectra of UCNP, UCNP@TiO_2_ composites and UCNP/TiO_2_ mixtures were examined under a 980 nm laser excitation (Fig. 2). Peaks of Tm ions at 291 nm, 345 nm and 361 nm in the UV region were assigned to the^1^I_6_-^3^H_6_,^1^I_6_-^3^F_4_, and^1^D_2_-^3^H_6_ transitions, respectively. And two emission peaks centered at 453 and 478 nm were attributed to^1^D_2_-^3^F_4_ and^1^G_4_-^3^H_6_ transitions of Tm ions, respectively. Interestingly, in the presence of TiO_2_, the intensities of the emissions at 291 nm nearly disappeared, 345 and 361 nm diminished, while the 453 and 478 nm emissions reduced slightly. Impressively, the intensity of emissions in the UV range (291, 345 and 361 nm) were decreased more greatly for UCNP@TiO_2_ composites than for the UCNP/TiO_2_ mixture.

**Figure 3:**
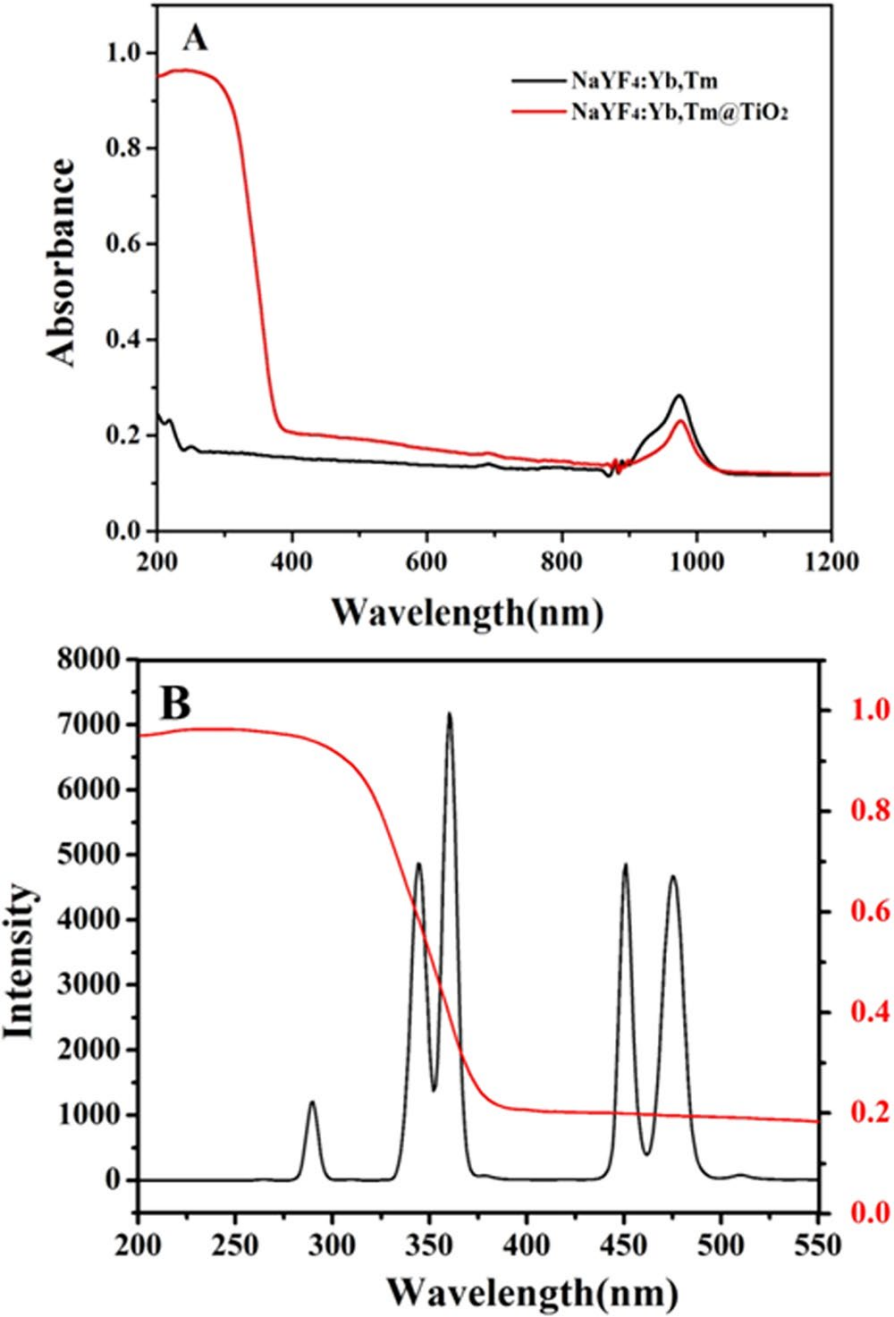
UV-vis-NIR absorbance of NaYF_4_:Yb, Tm and NaYF_4_:Yb, Tm@TiO_2_ (**A**) and the overlap spectra of NaYF_4_:Yb, Tm and TiO_2_ (**B**).

UV-vis-NIR absorption spectra of the as-prepared samples were shown in Fig. 3A. No absorption was found in the UV region for the bare UCNP. On the observation of the absorbance spectra of UCNP@TiO_2_, a sharp peak starting at 400 nm emerged, corresponding to TiO_2_ band gap absorption of 3.2 eV (~380 nm). Both bare UCNP and UCNP@TiO_2_ had an absorption peak at 980 nm, attributed to the absorption of Yb^3+^ ions. Therefore, we can also speculate UCNP@TiO_2_ core-shell nanocomposite had successfully prepared. The broad absorption band of TiO_2_ before 400 nm was overlapped with the^1^I_6_-^3^H_6_,^1^I_6_-^3^F_4_, and^1^D_2_-^3^H_6_ fluorescence emission peaks of UCNP under NIR irradiation (Fig. 3B).

On the basis of the above characterization and literature reports^30–32^, the energy migration between NaYF_4_:Yb, Tm and TiO_2_ coating which is resulted from the combined effect of radiation reabsorption and a fluorescence resonance energy transfer (FRET) process. While the energy migration of UCNP/TiO_2_ mixture, is only attributed to the radiation-reabsorption process. Benefiting from the synergy of FRET and radiation-reabsorption, the energy transfer efficiency of the NaYF_4_:Yb,Tm@TiO_2_ core-shell composite is higher than that of the physical mixture of NaYF_4_:Yb,Tm/TiO_2_ physical mixtures. It was especially useful to realize the NIR-driven photocatalytic activity of UCNP@TiO_2_ that will be investigated in the following section.

### Degradation of MC-LR with photocatalysis in different spectrums

**Figure 4:**
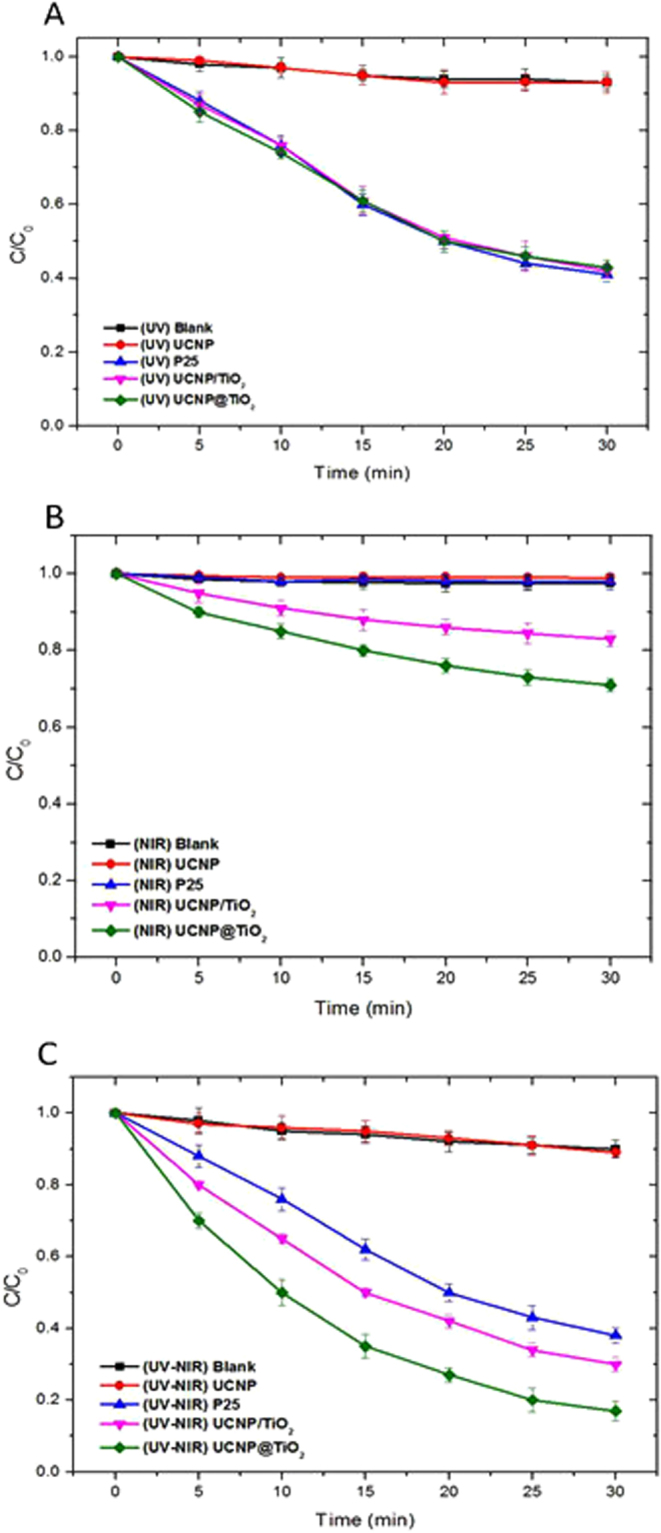
Comparisons of samples under different irradiation bands of Xe lamp: UV (**A**), NIR (**B**) and full spectrum (**C**).

In order to compare the catalytic efficiency of various photocatalysis in different spectrums, five experiment groups were set, including control group (no catalysis), bare UCNP, P25 (pure TiO_2_), UCNP/TiO_2_ and UCNP@TiO_2_ (all of them were 0.3 mg/mL). The pH of reaction system was 6.3. The concentration of MC-LR solution at each time interval was calculated using the area of the standard solution (10 μg/mL). The changes in concentration of MC-LR (C/C_0_) with different exposure times were plotted in Fig. 4. Figure 4A showed the concentration changes of MC-LR catalyzed by the as-prepared samples under UV (300-450 nm) irradiation as a function of the irradiation time. No MC-LR degradation was found without photocatalyst or just with UCNP. However, it was found that 55.9% MC-LR was degraded in 30 min by using UCNP@TiO_2_ as the photocatalyst while 58% by using UCNP/TiO_2_ and about 60% by using P25, indicating the UCNP@TiO_2_ and UCNP/TiO_2_ had the similar photocatalytic performance as P25 under UV band. This similar result should be attributed to the TiO_2_ shells formed on the particle surface, which can be activated by UV as well as solid TiO_2_ particles.

Interesting, distinct degradation of MC-LR was also observed when the photodegradation was carried out in the NIR band of the Xe lamp (780–2500 nm). As shown in Fig. 4B, the UCNP@TiO_2_ exhibited 30% photocatalytic activity towards the MC-LR, the degradation of UCNP/TiO_2_ is about 17%. In contrast, P25, control group and UCNP exhibited almost no activity, suggesting that the UCNP@TiO_2_ and UCNP/TiO_2_ can also work under the NIR-driven as photocatalysts, due to the energy transfer from UCNP to TiO_2_.

To further evaluate the effective contribution of photocatalysis in the simulate sunlight, the photocatalytic degradation activity of the photocatalyst were tested under full spectrum (300–2500 nm). As can be seen in Fig. 4C, 83% of MC-LR was degraded by using UCNP@TiO_2_, about 70% by using UCNP/TiO_2_ while 61% by using P25 within 30 min. Considering that photocatalyst (UCNP@TiO_2_, UCNP/TiO_2_, P25) showed no catalytic effect under visible light, the enhanced activity of UCNP@TiO_2_ under solar irradiation was mainly due to the utilization of NIR energy. This result was encouraging because it demonstrated that the introduction of NIR-to-UV upconversion agent into TiO_2_ can actually make the composite catalyst not only sensitive to NIR, but also to UV of solar energy, which will improve the utilization range of sunlight. More importantly, UCNP@TiO_2_ attached closely to each other and formed core-shell structure. Benefited from the effect of radiation reabsorption and FRET processes, more NIR photon had been used to activate TiO_2_ and high photocatalytic activity were obtained.

### Optimization of photocatalytic degradation by UCNP@TiO_2_

In order to obtain the optimal condition of photocatalytic activity, some important factors including the concentration of UCNP@TiO_2_, the system pH and reusability of samples were studied under UV to NIR irradiation. Firstly, the effect of concentration of photocatalyst was investigated with five different dosages (0.1, 0.2, 0.3, 0.4, and 0.5 mg/mL). The concentration of MC-LR solution was 10 μg/mL, pH was 6.3 and the results were shown in Fig. S4A. With the addition of photocatalyst, the photocatalytic efficiency of removing MC-LR increased. About 96% degradation can be achieved at the photocatalyst concentration of 0.4 mg/mL within 30 min of contacting time. However, when the concentration reached 0.5 mg/mL, the photocatalytic efficiency was reduced due to the shielding effect of UCNP@TiO_2_ to the Xe lamp. So the optimal photocatalyst concentration was 0.4 mg/mL.

The rate of photocatalytic reactions on UCNP@TiO_2_ photocatalysts has been found to be significantly influenced by pH. From the previous works^19^, the point of zero charge (pHzpc) of TiO_2_ was pH 6.20, below this level the surface was positively charged while above pH 6.20 TiO_2_ was negatively charged. MC-LR was positively charged below pH 2.10 and negatively charged above this point. In this study, effect of pH (2.0, 4.0, 6.3 and 8.3) on efficiency of photocatalytic degradation of MC-LR solutions (10 μg/mL) was investigated by using four sets of solutions at the photocatalyst concentration of 0.4 mg/mL (Fig. S4B). The results indicated that the optimal pH was 4.0 because of the attractive forces between the positively charged titania (TiOH^2+^) and the negatively charged toxin (MC-LRH^-^), which improved the adsorbent of MC-LR on the surface of UCNP@TiO_2_. Under the optimal condition, the efficiency of photocatalytic degradation of MC-LR can be improved to almost 100% within 30 min. To investigate the reusability of UCNP@TiO_2_, these NPs were recycled from the solution after one-time photocatalysis by centrifuge, washed with distilled water and dried in an oven overnight for next use. As shown in Fig. S4C, the UCNP@TiO_2_ had a good photocatalytic activity on MC-LR even after three circles. The efficiency of photocatalytic degradation of MC-LR can maintain above 85%. This result demonstrated that UCNP@TiO_2_ had an excellent chemical stability and can be employed as recyclable photocatalysts in many practical applications.

Furthermore, to quantitatively understand the reaction kinetics of MC-LR degradation in our experiments, we applied the pseudo-first-order model which was a widely used model to evaluate the photocatalytic degradation rate (Fig. S5A). Apparent degradation rate constants (k) were calculated by plots of -ln(C/C_0_) versus irradiation time and the values were shown in Table S1. The results indicated that all the experiment data fit a first-order kinetic model well, and UCNP@TiO_2_ significantly facilitated the degradation rate of MC-LR. Under the optimal condition, the observed rate constant (k) of UCNP@TiO_2_ was 0.147, which was approximately three times than that of P25 (Table S2). It verified that efficiency of photocatalytic degradation of UCNP@TiO_2_ was remarkable higher than pure TiO_2_ under the sunlight irradiation (Fig. S5B).

### Detection of photogenerated •OH radicals

**Figure 5:**
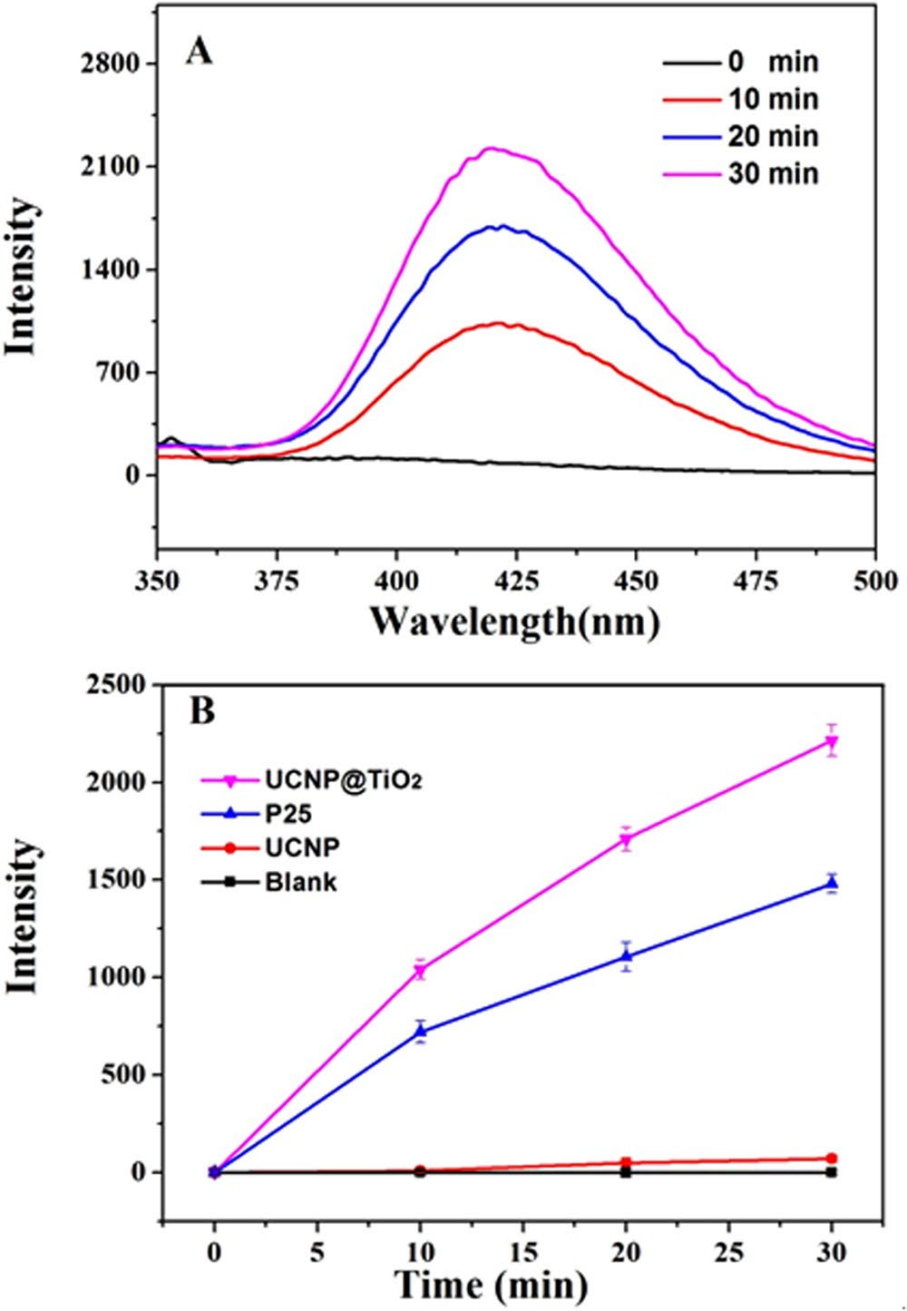
Fluorescence emission spectral of TAOH at different irradiation time (**A**), in the presence of different photocatalyst (**B**) under full spectrum.

Generally, hydroxyl radicals (•OH), a reactive oxygen species with strong oxidation ability, were considered mainly responsible for the degeneration of pollutant molecules in the photocatalytic reaction. In order to demonstrate the generation of •OH during the photocatalytic reaction, hydroxyl radicals trapping experiments were conducted. It is known that terephthalic acid (TA) itself does not emit fluorescence. However, the non-fluorescent TA can capture •OH to generate hydroxyterephthalic acid (TAOH), which emits fluorescence at 426 nm under the excitation of 320 nm UV light. Therefore, the formation of TAOH as well as the generation of •OH can be selectively and quantifiably detected by monitoring the emission intensity at 426 nm. As shown in Fig. 5A, the fluorescence intensity increased with the reaction time, indicating that more •OH was generated during the process. It also demonstrated that hydroxyl radicals, released from TiO_2_ shells, were the chief element for MC-LR degradation. It further illustrated that the content of •OH from UCNP@TiO_2_ was higher than that from P25 (Fig. 5B). Contrast with the photocatalytic efficiency of UCNP@TiO_2_ and P25, this result was maintained consistent.

### Analysis of intermediate products and proposed degradation pathway of MC-LR

**Figure 6:**
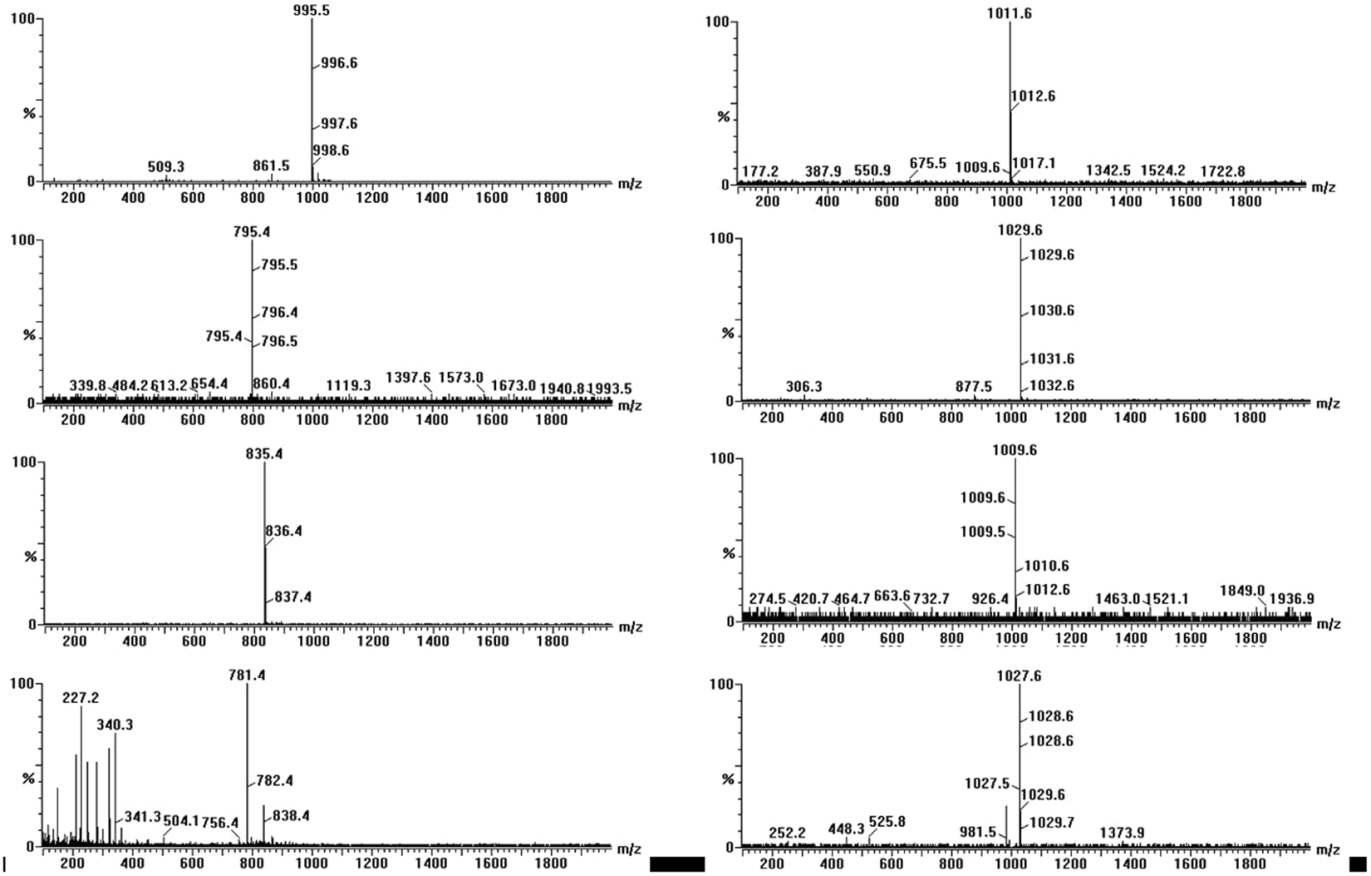
Mass spectrum of MC-LR and intermediates exposed to full spectrum.

**Figure 7:**
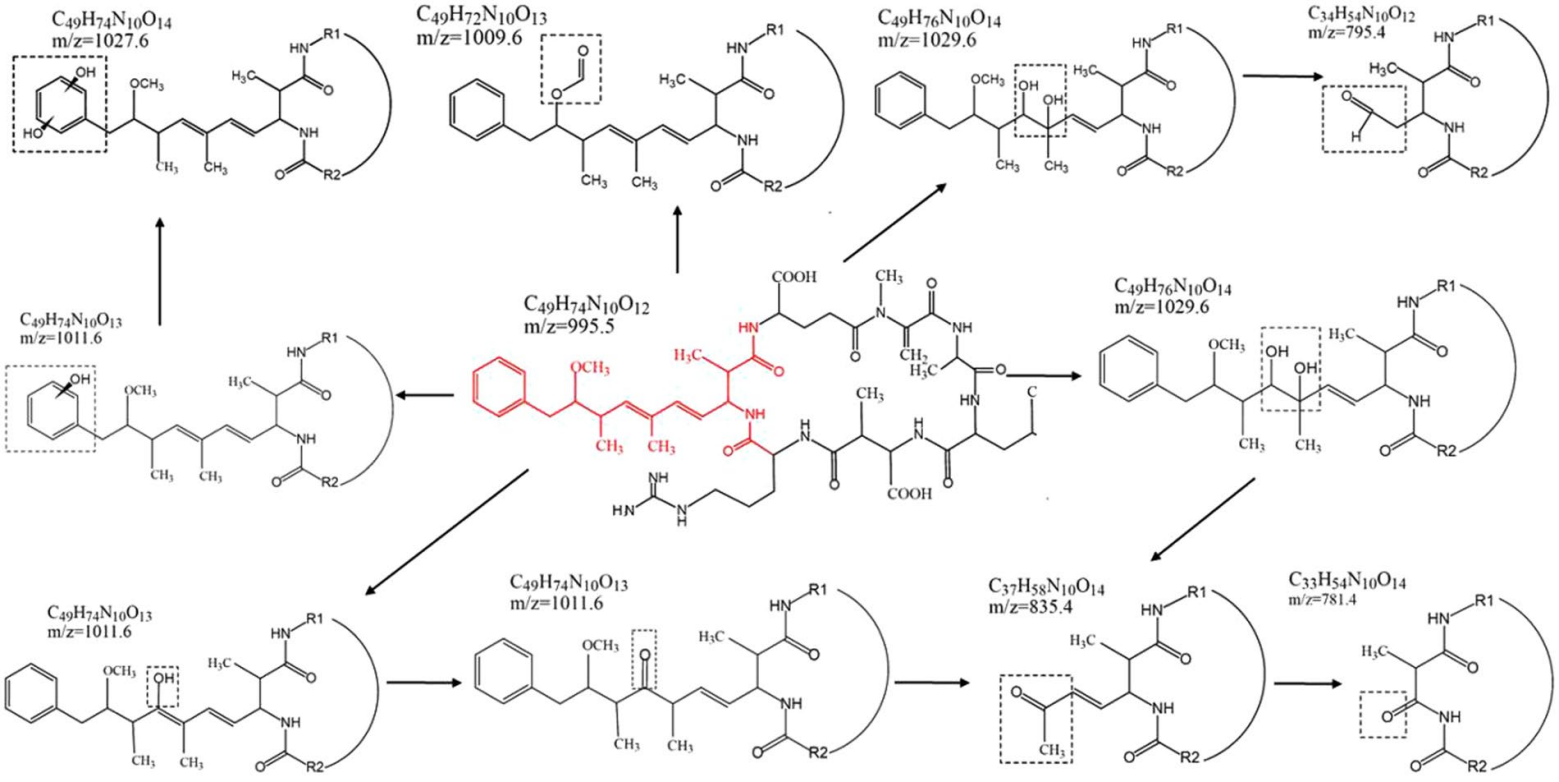
The proposed pathway and intermediates after hydroxyl radicals attacking Adda side chain of MC-LC.

The intermediate products of MC-LR degradation by using UCNP@TiO_2_ as the photocatalyst were investigated and performed by LC-MS/MS analysis. The HPLC peak at about 8.70 min of total ion chromatogram (TIC) and selective ion flow diagram in the positive ESI mode were shown in Fig. S6, which exhibited obvious decreased resulting photocatalytic degradation of MC-LR and appeared several peaks of intermediate products under simulate sunlight irradiation. As shown in Fig. 6, each bar in mass spectrum represents an ion having a specific mass-to-charge ratio (m/z). Due to the m/z value is equivalent to mass itself; the intermediate products can be determined by the m/z value. Overall, seven intermediates (m/z = 1029.6, 795.4, 835.4, 1009.6, 1011.6, 1027.6 and 781.4) were observed via photocatalytic degradation of MC-LR. The intermediates with m/z = 1029.6 had 34 Da of difference in molecular weight to the MC-LR (m/z 995.5). It was possible generated from the dihydroxylation of either C4-C5 or C6-C7 double bonds of the Adda side chain. Firstly, •OH was added to one end of the above double bonds through the attack of side chain and formed allyl radical. Subsequently, •OH was reacted with allyl carbocation and generated di-hudroxyl MC-LR. The intermediates with m/z = 795.4 was a type of aldehyde-derivative, resulting from the oxidized oxidative induced bond cleavage between C4-C5 of the Adda chain of the above dihydroxylation products. Similarly, the intermediates with m/z = 835.4 was a type of ketone-derivative, which was formed from the split of the C6-C7 in the Adda chain of the above dihydroxylation products. The intermediates with m/z = 781.4 had 54 Da of difference in molecular weight to the m/z 835.4. It was possibly resulted from the decarboxylation of m/z 835.4. The intermediates with m/z = 1009.6, a type of formate ester-derivative, was likely formed by the hydrogen abstraction mechanism on the methoxy group of Adda by •OH. The intermediates with m/z = 1011.6 had 16 Da of difference in molecular weight to the MC-LR. It was probably resulted from the hydroxyl substitution on the double bonds of Adda, that is, the hydrogen atom of C7 was substituted by •OH and generated enol form MC-LR. However, the enol form MC-LR was unstable, the ketone-derivative of m/z = 835.4 was thus generated with the split of the C6-C7. In addition, the intermediates with m/z = 1011.6 was also probably generated from hydroxyl substitution on the aromatic ring of Adda. The hydrogen atom on the aromatic ring was substituted by •OH. The intermediates with m/z = 1027.6 also had 16 Da of difference in molecular weight to m/z = 1011.6. Electrophilic substitution with •OH radicals was presumed to occur at the ortho and para positions. The m/z = 1027.6 was the second hydroxylation resulted from the first aromatic hydroxylation thickening the electron density on the aromatic ring. The mechanism of photocatalytic degradation for MC-LR was probably speculated in Fig. 7. This is mainly because Adda side chain of MC-LC was attacked and broken by hydroxyl radicals.

### Evaluation of toxicity of degradation products

While special attention is paid to the degradation efficiency of MC-LR, the toxicity of MC-LR after degradation should also be concerned with. It was reported that the toxicity of MC-LR was mainly conjugated diene structures of Adda side chain which inhibited the activity of protein phosphatases 1 and 2. That affected the demethylation function of protein and resulted in disorder of intracellular metabolism. The degradation products of MC-LR via UCNP@TiO_2_ were identified and mainly divided into 3 categories: Adda side chain modified (m/z = 1011.6, 1027.6, 1029.6); Adda side chain removal of methoxyl group (m/z = 1009.6) and Adda side chain cleavage (m/z = 795.4, 835.4, 781.4). In the extrapolation of the above reaction pathways, there are several ways to destroy Adda side chain: double bond hydroxylation and oxidative cleavage, hydrogen abstraction, hydroxyl substitution on benzene ring and so on. Since the Adda group is essential for the expression of toxicity of MC-LR, these processes can change the geometric conformation of Adda group, which can prevent their effective binding to protein phosphatase and eliminate their toxicity significantly. In previous works, Antoniou *et al*.^33^ and Fotiou *et al*.^34^ carried out photocatalytic degradation of MC-LR with TiO_2_ photocatalytic films and graphene oxide-TiO_2_ composite respectively. The seven degradation products of our study were also reported in these two researches (m/z 1029.5, m/z 1011.5, m/z 1009.5 and m/z 1027.5 in Antoniou’s study, m/z 795, m/z 835.5 and m/z 781.5 in Fotiou’s study). Both of them evaluated the toxicity of degradation products by using the protein phosphatase inhibition assay (PPIA) via a colorimetric method. The results showed that the toxicity of the products was completely removed as soon as the molecule of MC-LC was degraded to oxidized products. Meanwhile, Lawton^35^ used TiO_2_ to destroy MC-LR in aqueous solution and tested the toxicity of degradation products by using an invertebrate bioassay. The results indicated that the degradation of MC-LR was accompanied by a reduction of its toxicity, due to the actually non-toxic of the degradation products. The process of MC-LR degradation by UCNP@TiO_2_ in our work is corresponding to the above studies, resulting from the hydroxyl radical. Thus, it was predicted that this advanced photocatalyst was environmentally friendly and the toxicity of intermediates was lower and even nontoxic.

## Discussion

**Figure 8:**
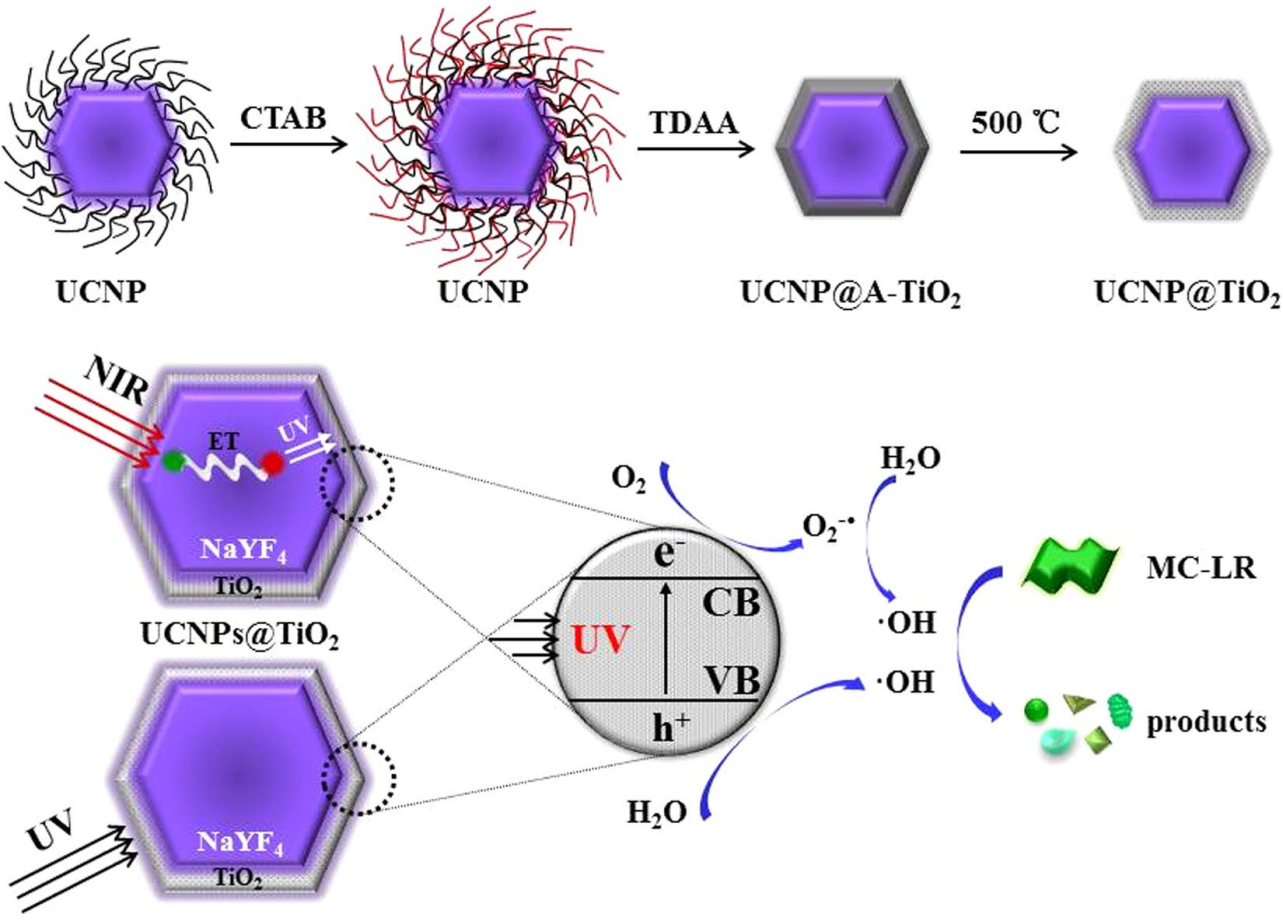
Schematic diagram for illustrating the synthesis and photocatalytic process via the NaYF_4_:Yb, Tm UCNP@TiO_2_ photocatalyst under imulated solar lights irradiation.

In our proposal, a novel core-shell NaYF_4_:Yb, Tm UCNP@TiO_2_ composite was designed and employed as the photocatalyst for MC-LR degradation. The synthetic strategy and photocatalysis were illustrated in Fig. 8. Firstly, UCNP were synthesized via hydrothermal method by oleic acid capped. Then, the as-prepared UCNP were modified by CTAB to form the hydrophilic UCNP. TDAA were used to coating a crystalline anatase TiO_2_ shell on the surface of UCNP though the method similar to Stöber route and the annealing process. The sunlight photocatalytic activity of composite photocatalysts was approximately 3 times than pure TiO_2_ and approaching 100% of MC-LR was photodegraded within 30 min. The MC-LR degradation rates in other articles compared with our work were listed in the Table S4 in the supporting information. The greatly enhanced photocatalytic activity of UCNP@TiO_2_ under ultraviolet-NIR-driven can be attributed to several reasons: NaYF_4_:Yb, Tm UCNP acted as a medium for converting NIR to UV and visible light via multiphoton upconversion processes. Much more reactive oxygen species in the photocatalytic reaction were generated benefited from the synergistic effects by UV and NIR irradiation. On the basis of the above nano-catalyst material, a possible photocatalytic process for MC-LR degradation under simulate sunlight irradiation was deduced. The LC-MS/MS analysis confirmed the photodegradation of MC-LR and investigated the degradation products, which gave *m*/*z* values of 1029.6, 795.4, 835.4, 1009.6, 1011.6, 1027.6 and 781.4, suggesting that MC-LR was reduced by photodegradation and the seven intermediates formed, respectively. The reaction mechanism was hydroxylation on the aromatic ring of Adda and on the diene bonds of Adda. Due to the destruction of Adda construction, the intermediates were nontoxic. In conclusion, the upconversion nanoparticle-TiO_2_ activated by cooperation of UV and NIR light, can provide important inspirations and be considered as an ideal and effective photocatalysts for mycotoxin detoxification and food security applications.

## Methods

### Synthesis and modification of UCNP

NaYF_4_:20%Yb, 0.5%Tm nanoparticle were synthesized using a user-friendly procedure as our previously reported^36^. In brief, YCl_3_·6H_2_O, YbCl_3_·6H_2_O and ErCl_3_·6H_2_O (Ln = Y:79.5%, Yb:20%, Er:0.5%) were mixed with 4 mL oleic acid and 16 mL octadecene in a 100 mL flask, heated to 160 °C to form a homogeneous solution, and then cooled to room temperature. A total of 10 mL of a methanol solution containing 4 mmol of NH_4_F and 2.5 mmol of NaOH was slowly added to the flask. The solution was stirred, and the temperature was raised to evaporate the methanol, then the solution was degassed at 80 °C, heated to 300 °C and maintained for 1 h under an argon atmosphere. After the solution was cooled, nanocrystals were precipitated from the solution and washed with ethanol three times. The prepared nanoparticles were dispersed in 10 mL of cyclohexane, reaching a concentration of 0.1 M. To modify the particle surface, a reverse-micelle approach was employed. Typically, 0.05 g cetyltrimethyl ammonium bromide (CTAB) and 1 mL of UCNP solution were added to 20 mL deionized water with vigorous stirring in a flask. Once a milky solution was formed, the flask was then move into a water-bath (80 °C) to slowly evaporate cyclohexane. The milky solution gradually became transparent, and cooled down to room temperature. The CTAB modified UCNP (NaYF_4_:Yb,Tm/CTAB) were subsequently collected from the solution by centrifuging at a speed of 8000 rpm. After washing with water twice, the precipitations were finally re-dispersed in 10 mL isopropanol.

### Synthesis of NaYF_4_:Yb,Tm@TiO_2_ NPs

NaYF_4_:Yb,Tm@TiO_2_ NPs were obtained by coating a TiO_2_ layer on the surface of NaYF_4_:Yb,Tm/CTAB NPs. Typically, 10 mL of NaYF_4_:Yb,Tm/CTAB NPs solution (in isopropanol), 0.3 mL ammonia (28 wt%) and 2.5 mL water were mixed in a flask under magnetic stirring. Subsequently, 36 μL of titanium diisopropoxide bis (acetylacetonate) (TDAA) in 10 mL isopropanol was slowly injected into the solution. The mixed solution was then aged in the flask under stirring for 12 h at room temperature. Core–shell NaYF_4_:Yb,Tm@A-TiO_2_ (amorphous TiO_2_) NPs were then collected from the solution by centrifuging at a speed of 6000 rpm. After washing with ethanol and isopropanol twice, the precipitations were dried in a vacuum. To achieve a crystalline anatase TiO_2_ shell, the products were finally annealed at 500 °C for 3 h in an oven under an atmosphere of air.

### Photocatalytic activity measurements

In a typical experiment, the photocatalyst (UCNP@TiO_2_, P25) was dispersed into a glass tube containing aqueous solution of MC-LR (10 μg/mL) with constant stirring and then kept in the dark 2 h prior to irradiation for establishing adsorption-desorption equillibrium between MC-LR and the surface of photocatalyst. Subsequently, 500 W Xe lamp with different bands was used as the UV (300–400 nm), NIR (780–2500 nm) and full spectrum irradiation source (300–2500 nm). After irradiation for a designated time, 0.3 mL of MC-LR solution was taken out for determining by HPLC analysis after centrifuging to remove catalyst. Following, some important factors for better degradation efficiency including the concentration of UCNP@TiO_2_, the system pH and reusability of samples were investigated.

### Identification of intermediate products

For the identification of MC-LR intermediates, a solution of 5 mL containing 10 μg/mL MC-LR and 0.4 mg/mL UCNP-TiO_2_ was irradiated and the some samples were taken out at certain time intervals during the process. Samples were then centrifuge and analyzed by LC-MS/MS using electro-spray ionization (ESI) source in the positive mode.

## Electronic supplementary material

supporting information
