# Supplementary material for: Photocatalytic degradation of microcystin-LR with a nanostructured photocatalyst based on upconversion nanoparticles@TiO2 composite under simulated solar lights
Source: Sci Rep. 2017 Oct 31;7:14435. doi: 10.1038/s41598-017-14746-6 (PMC5663843; doi:10.1038/s41598-017-14746-6)
Supplement: Supplementary file 1 — supporting information [file 41598_2017_14746_MOESM1_ESM.pdf]

Photocatalytic degradation of microcystin-LR with a nanostructured photocatalyst based on upconversion nanoparticles-TiO_2 composite under simulated solar lights

Shijia Wu[1,2*], Jiajia Lv[2], Fang Wang[2], Nuo Duan[1,2], Qian Li[2], Zhouping Wang[1,2*]

[1] *State Key Laboratory of Food Science and Technology, Jiangnan University, Wuxi 214122, China*

[2] *School of Food Science and Technology, Jiangnan University, Wuxi 214122, China*

* Corresponding author. Tel/Fax: +86 510 8591 7023. E-mail: wusj1986@163.com; wangzp@jiangnan.edu.cn.

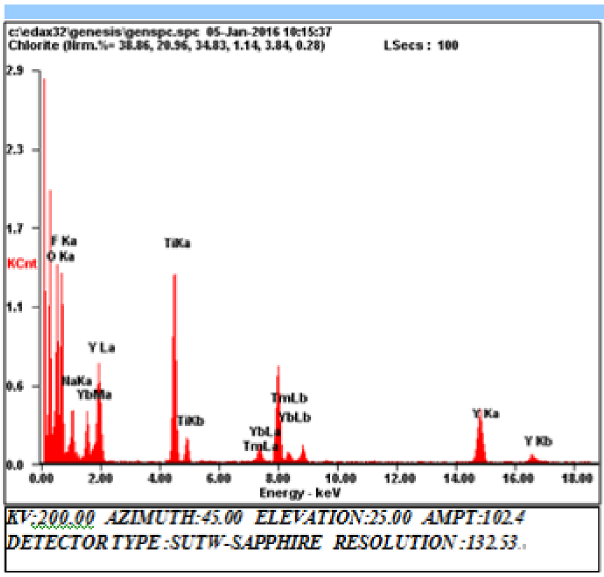

Elem	*Weight %*	*Atomic %*
O K	17.20	31.80
F K	27.20	42.30
NaK	03.40	04.40
TiK	21.40	13.20
TmL	01.10	00.20
YbL	11.60	02.00
Y K	18.20	06.00

Figure S1. Composition analysis by EDS of $NaYF_4$:Yb, Tm@TiO_2.

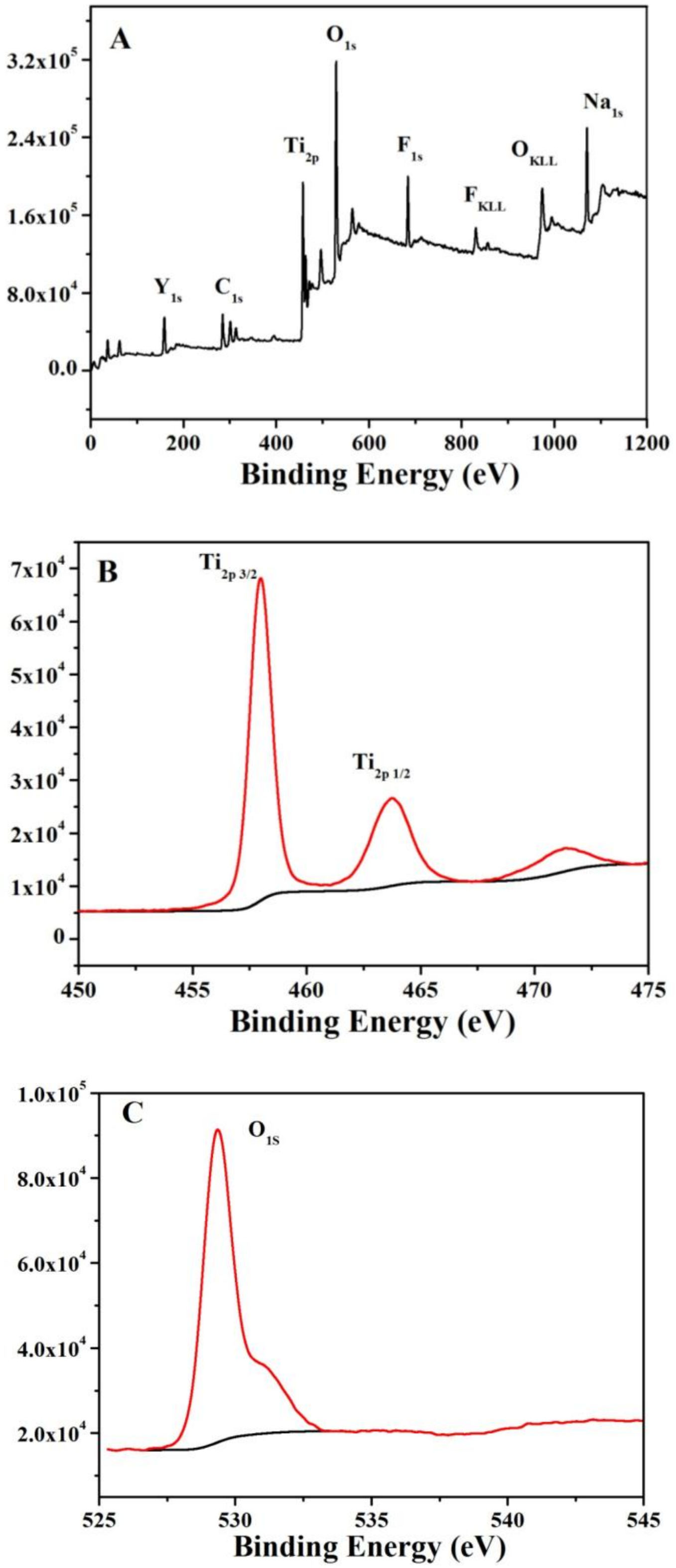

Figure S2. XPS spectra of $NaYF_4$:Yb, Tm@TiO_2 (A), element Ti (B) and element O (C).

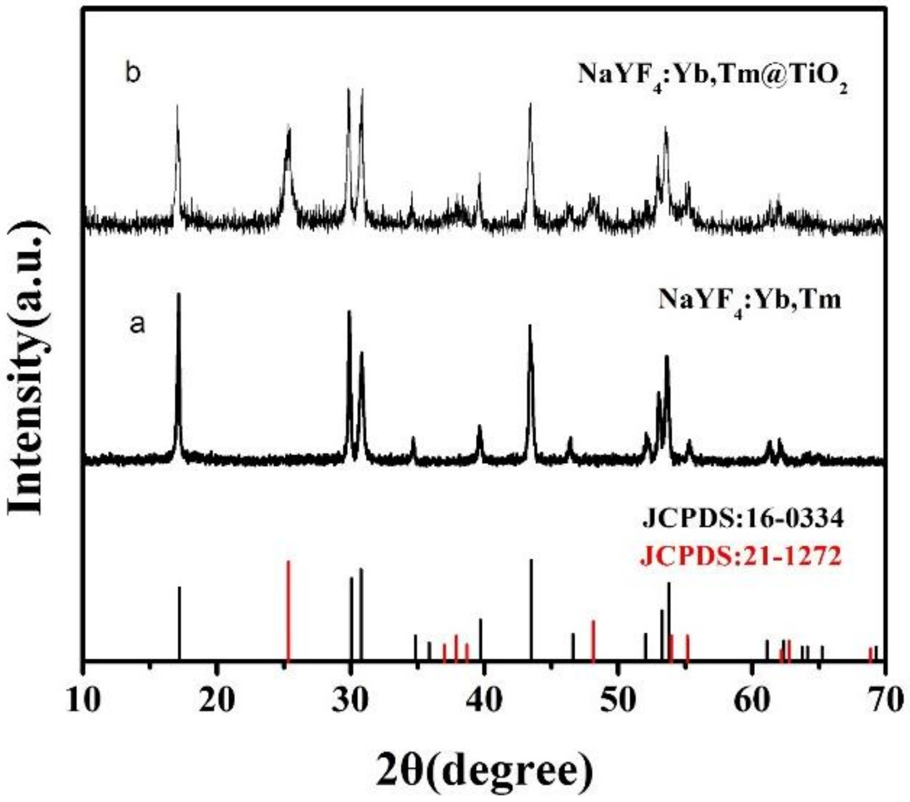

Figure S3. XRD patterns of $NaYF_4$:Yb, Tm (a) and $NaYF_4$:Yb, Tm@TiO_2 nanoparticles (b).

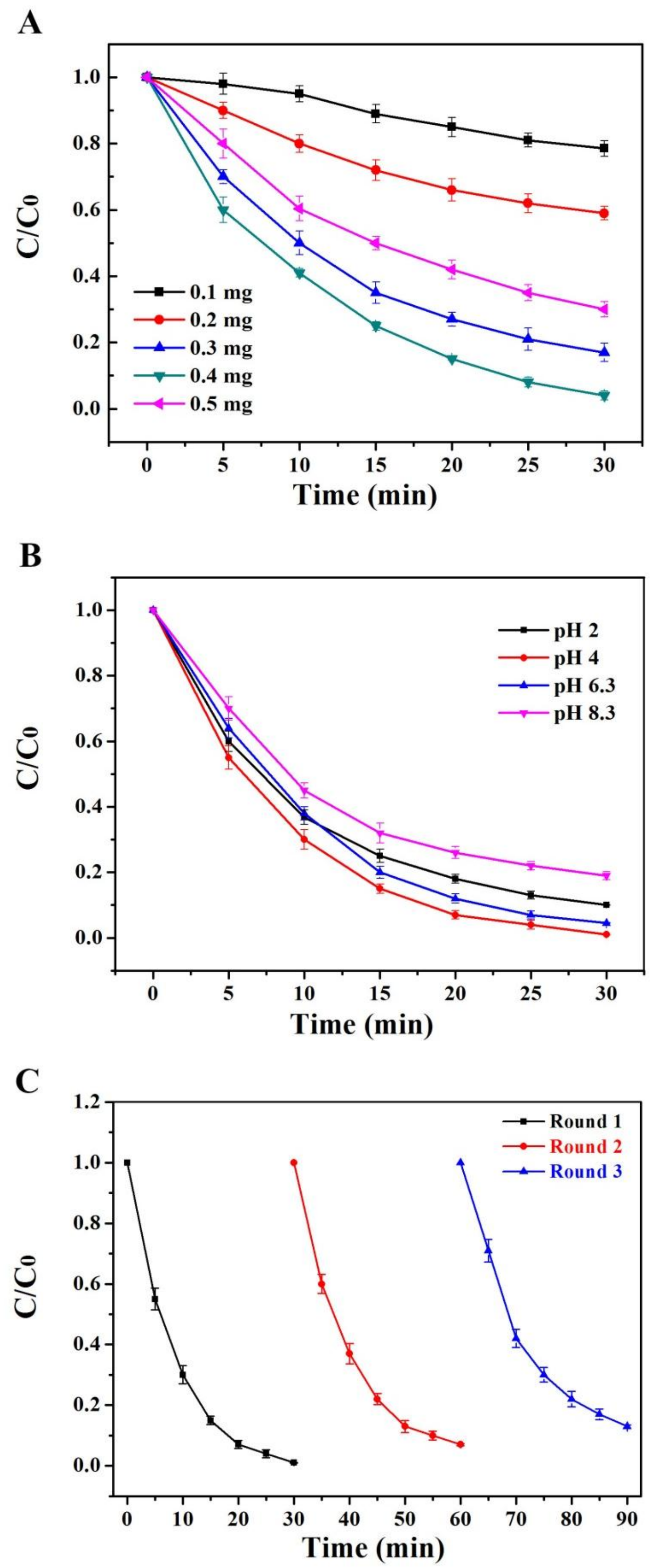

Figure S4. Photocatalytic degradation of MC-LR in the presence of different initial concentration of $NaYF_4$:Yb, Tm@TiO_2 under full spectrum (A), Photocatalytic degradation of MC-LR at different pH under full spectrum (B), Recyclability of the NaYF4:Yb, Tm@TiO_2 nanoparticles for the degradation of MC-LR via centrifugation (C).

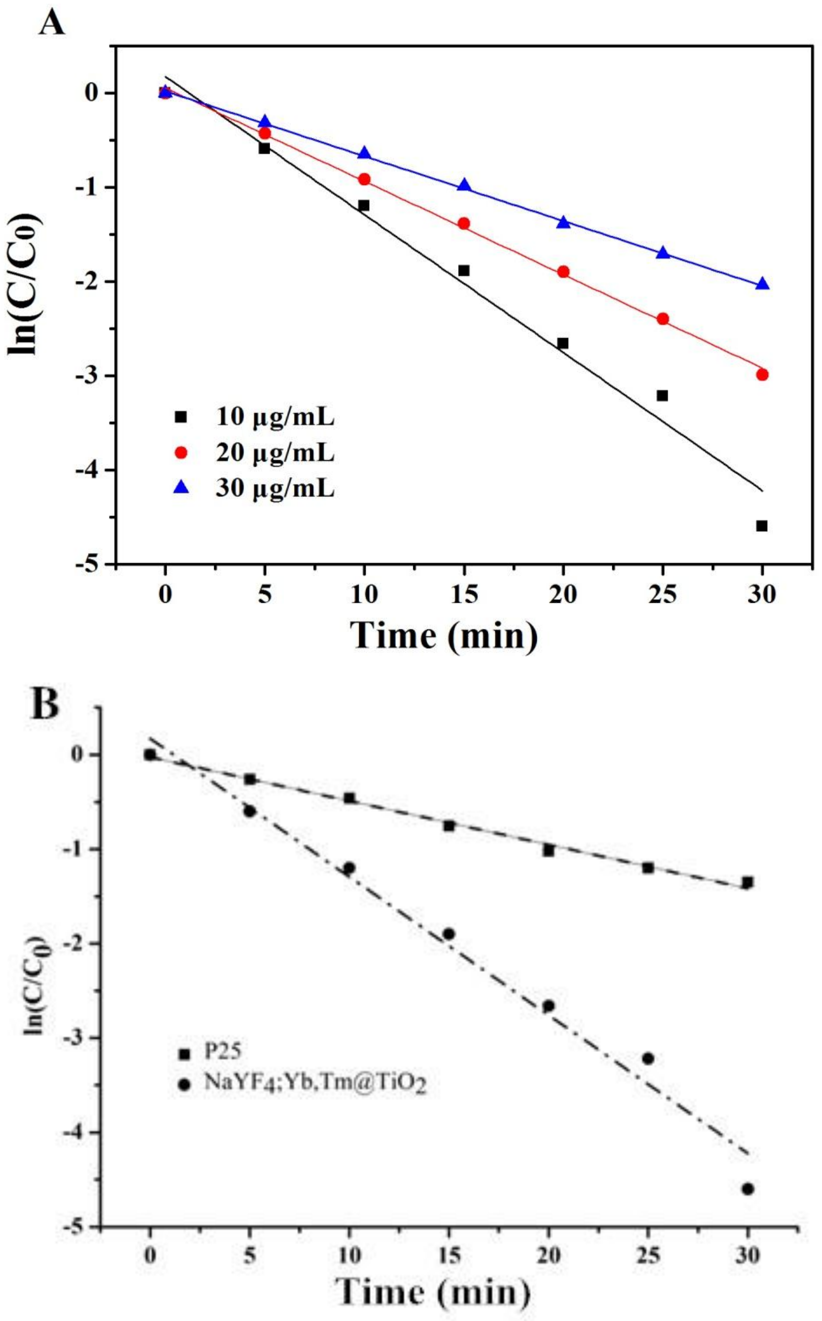

Figure S5. Kinetic curve of UCNP@TiO_2 with different MC-LR concentration under full spectrum (A), Pseudo-first-order kinetics of the degradation of MC-LR with different photocatalysts (B)

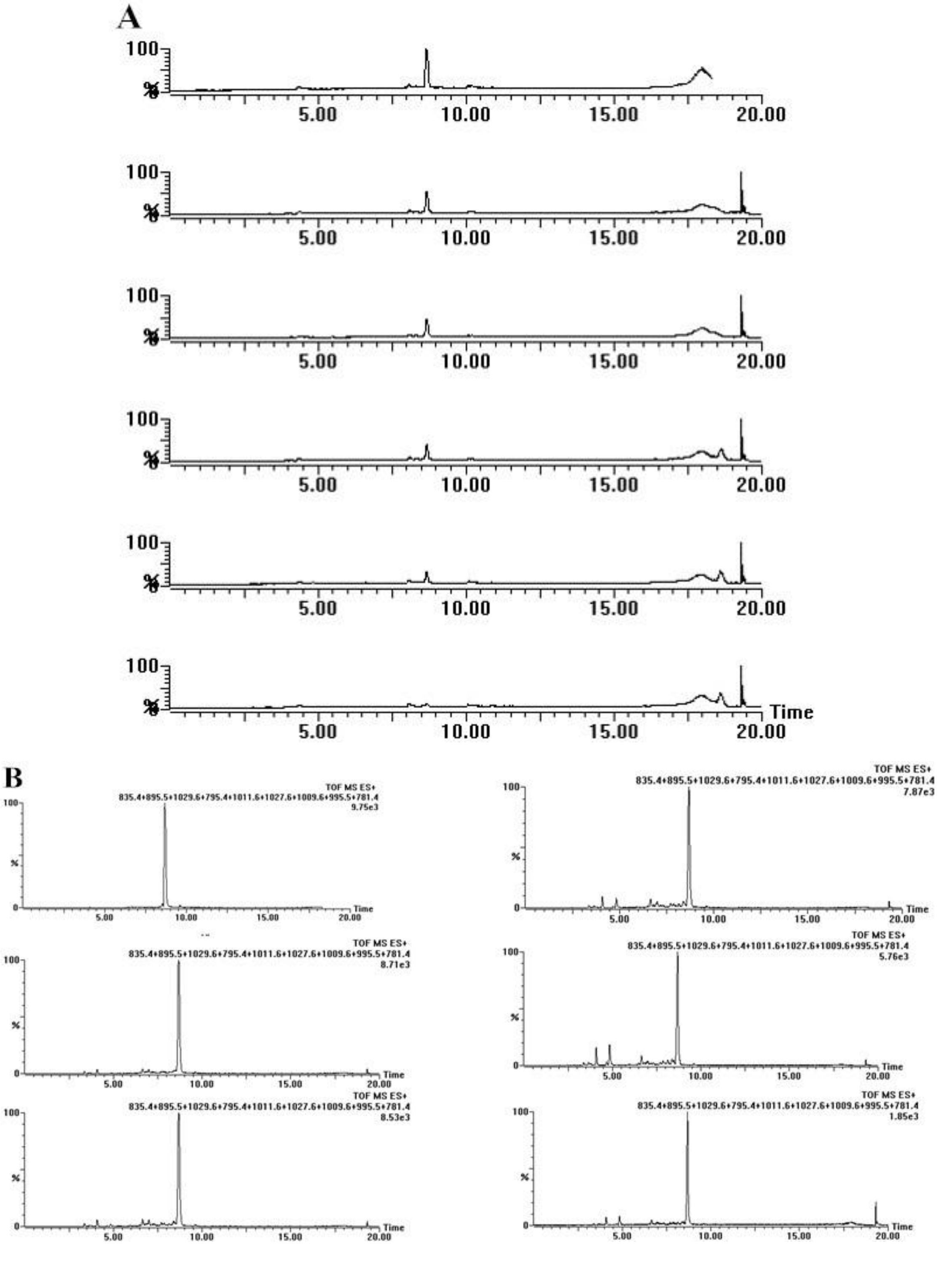

Figure S6. The total ion chromatogram (TIC) (A) and the selective ion flow diagram (B) of the MC-LR and intermediates vs. time.

Table S1. Kinetic parameters of photocatalytic degradation of MC-LR at varying concentrations

C_0(μg/mL)	Pseudo-first-order kinetics	k(min^{-1})	R^2
10	$\ln(C_0/C)$=-0.147 t + 0.172	0.147	0.980
20	$\ln(C_0/C)$=-0.099 t + 0.060	0.099	0.997
30	$\ln(C_0/C)$=-0.069 t + 0.019	0.069	0.999

Table S2. Kinetic parameters of degradation of MC-LR with different photocatalysts

Photocatalysis	Pseudo-first-order kinetics	k(min^{-1})	R^2
$NaYF_4$:Yb, Tm@TiO_2	$\ln(C_0/C)$=-0.147 t + 0.172	0.147	0.980
P25	$\ln(C_0/C)$=-0.046 t - 0.025	0.046	0.991

Table S3. Intermediates detected from the degradation of MC-LR by $NaYF_4$:Yb, Tm@TiO_2

No	Peak (m/z)	Reserve Time (min)
1	995.5	8.676
2	781.4	3.643
3	795.4	3.375
4	835.4	4.083
5	1009.6	9.609
6	1011.6	6.509
7	1027.6	8.814
8	1029.6	6.647

Table S4. The comparison of degradation efficiency of the current methods for MC-LR

Methods and reagents	Reagent dosage (mg/mL)	Spectra	Intial MC-LR (μg/mL)	Degradation rate	Reaction time (min)	Reference
O_3	1.5	——	50	70%	1	Heng-Feng Miao, et al. [1]
H_2O_2	4.0	UV-B (312 nm)	0.1	~100%	120	Moon, Bo-Ram, et al. [2]
Fenton reagent (Fe(Ⅱ)/H_2O_2)	5×10^{-3} Fe(II) and 5×10^{-3} H_2O_2	——	0.2	76.77%	30	Park, Jeong-Ann, et al. [3]
O_3/ H_2O_2	0.1×10^{-3} O_3 and 0.1×10^{-4} H_2O_2	——	1	95%	1.3	Al Momani, Fares, et al. [4]
Mn/Ti-doped carbon xerogel	1 wt% Ti and 0.5 wt% Mn	UV	10	88.6%	6	Xin, Qing, et al. [5]
3D interconnected mesoporous TiO_2	0.3	UV-C (λ = 254 nm)	10	100%	17.5	Dong, Weiyang, et al. [6]
TiO_2 nano-film	38×51×1.5 mm	UV-A (λmax = 365nm)	0.02	95%	120	Feng, Xiaogang, et al. [7]
TiO_2–graphene@Fe3O4	0.5	UV-A (λmax = 365nm)	0.5	~100%	30	Liang, Yulu, et al. [8]
TiO_2 coated glass microspheres	58.8	UV–vis (330-450nm)	10	~100%	8	Pestana, Carlos J., et al. [9]
TiO_2 (Degussa P-25)	1.0	UV- vis (330-450nm)	1000	100%	45	Liu, Iain, et al. [10]
NF-TiO_2	0.5	UV–vis	0.5	~100%	30	Pelaez, Miguel, et al. [11]
As-synthesized Ag_3PO_4 photocatalyst	26.67	visible light	9.06	99.98%	300	Sui, Xin, et al. [12]
C, N and S doped mesoporous anatase–brookite TiO_2	0.5	visible light	0.5	~100%	300	El-Sheikh, Said M., et al. [13]
Graphene oxide-TiO_2 composite	0.2	solar light	10	97%	60	Fotiou, Theodora, et al. [14]
Nitrogen doped TiO_2	0.2	solar light	10	100%	240	Triantis, T. M., et al. [15]
UCNP@TiO_2	0.4	solar light	10	100%	30	This study

HPLC conditions

Separation was achieved on an BEH C18 analytical column (150 mm×2.1 mm，1.7 μm) and under the column temperature of 45 °C. The mobile phase A is acetonitrile, and methanol is for mobile phase B. The gradient program was 0-15 min from 10% A to 70% A, 15-18 min from 100% A to 70% A, and 18-18.1 min from 100% A to 10% flowing at 0.8 mL/min. The UV detector was set at 238 nm.

Mass spectrometer conditions

Quadrupole time-of-flight mass spectrometer was used for detection with an electro-spray ionization (ESI) source in the positive mode. Ion source temperature is 100 ℃, capillary voltage 3.5 V, vaporizer temperature 400 ℃. The mass range acquired was from 100 to 2000 m/z.

Comparison of the current degradation methods

The MC-LR degradation rates in other articles compared with our work were listed in the Table S4. Different types of methods and several nanocomposites based on TiO_2 were included. Some chemical reagents were used to degrade MC-LR, mainly including H_2O_2, O_3. High dose of H_2O_2 or O_3 was needed in order to high efficiency. In some reports, synergy of two or three chemical reagents maybe could improve the efficiency. However, chemical reagents cannot oxidize all MC-LR in water owing to its poor solubility. In addition, such chemically intensive technology may form harmful even carcinogenic disinfection by-product. High costs associated with chemical processes generally make them unaffordable in practical treatments. Photocatalytic oxidation technology has attracted increasing attention because of its low energy consumption, simple operation and nontoxicity. In particular, TiO_2-based photocatalysis is one of the most common technologies. Due to the wide band gap of TiO_2, conventional methods of photocatalytic degradation were still restricted by UV or visible radiation. To achieve high degradation efficiency, these processes required longer reaction time and more dosage of photocatalysts. Some advanced methods

were used to degrade MC-LR by solar light, because TiO_2 doped with graphene oxide or nitrogen extended the light adsorption. In this study, UCNP@TiO_2 photocatalysts were used to absorb the NIR energy of solar lights as driving source for photocatalysis besides the UV light. Compared with the previous methods, this study exhibits a higher degradation rate, time saving, less dosage usage and environmentally sustainable.

References

[1] Miao, H. F., Qin, F., Tao, G. J., Tao, W. Y. & Ruan, W.Q. Detoxification and degradation of microcystin-LR and -RR by ozonation. *Chemosphere*. **79**, 355–361 (2010).

[2] Moon, B. R., Kim, T. K., Kim, M. K., Choi, J. & Zoh, K. D. Degradation mechanisms of Microcystin-LR during UV-B photolysis and UV/H_2O_2 processes: Byproducts and pathways. *Chemosphere*. **185**, 1039-1047 (2017).

[3] Park, J. A., Yang, B., Park, C., Choi, J. W., van Genuchten, C. M. & Lee, S. H. Oxidation of microcystin-LR by the Fenton process: Kinetics, degradation intermediates, water quality and toxicity assessment. *Chem Eng J*. **309**, 339-348 (2017).

[4] Al Momani, F., Smith, D. W. & EI-Din, M. G. Degradation of cyanobacteria toxin by advanced oxidation processes. *J Hazard Mater*. **150**, 238-249 (2008).

[5] Xin, Q., Zhang, Y., Li, Z. J., Lei, L. C. & Yang, B. Mn/Ti-doped carbon xerogel for efficient catalysis of microcystin-LR degradation in the water surface discharge plasma reactor. *Environ Sci Pollut R*. **22**, 17202-17208 (2015).

[6] Dong, W. Y., Yao, Y. W., Li, L., Sun, Y. J., Hua, W. M., Zhuang, G. S., Zhao, D. Y., Yan, S. W. & Song, W. H. Three-Dimensional Interconnected Mesoporous Anatase TiO2 Exhibiting Unique Photocatalytic Performances. *Appl Catal B: Environ*. **217**, 293-302 (2017).

[7] Fieng, X. G., Rong, F., Fu, D. G., Yuan, C. W. & Hu, Y. Photocatalytic degradation of trace-level of Microcystin-LR by nano-film of titanium dioxide. *Chinese Sci Bull*. **51**, 1191-1198 (2006).

[8] Liang, Y. L., He, X. W., Chen, L. X. & Zhang, Y. K. Preparation and characterization of TiO_2–Graphene@ Fe_3O_4 magnetic composite and its application in the removal of trace amounts of microcystin-LR. *RSC Adv*. **4**, 56883-56891 (2014).

[9] Pestana, C. J., Edwards, C., Prabhu, R., Robertson, P. K. J. & Lawton, L. A. Photocatalytic degradation of eleven microcystin variants and nodularin by TiO2 coated glass microspheres. *J Hazard Mater*. **300**, 347-353 (2015).

[10] Liu, I., Lawton, L. A., Cornish, B. & Robertson, P. K. J. Mechanistic and toxicity studies of the photocatalytic oxidation of microcystin-LR. *J Photoch Photobio A*. **148**, 349-354 (2002).

[11] Pelaez, M., Falaras, P., Likodimos, V., O'Shea, K., de la Cruz, A. A., Dunlop, P. S. M., Byrne, J. A. & Dionysiou, D. D. Use of selected scavengers for the determination of NF-TiO2 reactive oxygen species during the degradation of microcystin-LR under visible light irradiation. *J Mol Catal A-Chem*. **425**, 183-189 (2016).

[12] Sui, X., Wang, X. R., Huang, H. H., Peng, H. H., Peng, G. T., Wang, S. B. & Fan, Z. Q. A novel photocatalytic material for removing microcystin-LR under visible light irradiation: degradation characteristics and mechanisms. *PloS one*. **9**, e95798 (2014).

[13] El-Sheikh, S. M., Zhang, G. S., EI-Hosainy, H. M., Ismail, A. A., O'Shea, K. E., Falaras, P., Kontos, A. G. & Dionysiou, D. D. High performance sulfur, nitrogen and carbon doped mesoporous anatase–brookite TiO_2 photocatalyst for the removal of microcystin-LR under visible light irradiation. *J Hazard Mater*. **280**, 723-733 (2014).

[14] Fotiou, T., Triantis, T. M., Kaloudis, T., Pastrana-Martinez, L. M., Likodimos, V., Falaras, P., Silva, A. M. T. & Hiskia, A. Photocatalytic degradation of microcystin-LR and off-odor compounds in water under UV-A and solar Light with a nanostructured photocatalyst based on reduced graphene oxide-TiO2 composite. Identification of Intermediate Products. *Ind. Eng. Chem. Res.* **52**, 13991-14000 (2013).

[15] Triantis, T. M., Fotiou, T., Kaloudis, T., Kontos, A. G., Falaras, P., Dionysiou, D. D., Pelaez, M. & Hiskia, A. Photocatalytic degradation and mineralization of microcystin-LR under UV-A, solar and visible light using nanostructured nitrogen doped TiO_2. *J Hazard Mater*. **211**, 196-202 (2012).
